# A Clinical Significance of Fungal Infections in Diabetic Foot Ulcers

**DOI:** 10.7759/cureus.26872

**Published:** 2022-07-14

**Authors:** Sowmya Kandregula, Aparna Behura, Chinmaya R Behera, Dipti Pattnaik, Amaresh Mishra, Bandita Panda, Subrat Mohanty

**Affiliations:** 1 Surgery, Kalinga Institute of Medical Sciences, Bhubaneswar, IND; 2 Pathology, Kalinga Institute of Medical Sciences, Bhubaneswar, IND; 3 Microbiology, Kalinga Institute of Medical Sciences, Bhubaneswar, IND; 4 Research and Development, Kalinga Institute of Medical Sciences, Bhubaneswar, IND; 5 Surgery (Pediatric Surgery), Kalinga Institute of Medical Sciences, Bhubaneswar, IND

**Keywords:** candida albicans, clinical outcome, culture, fungal infection, diabetic foot ulcer

## Abstract

Background: Diabetic foot ulcers (DFUs) are the most common and serious complications in uncontrolled diabetes. Infections are predominantly polymicrobial, with aerobic Gram-positive, anerobic, and fungal infections. Early detection of fungal infection and initiation of appropriate treatment in DFUs may lead to better healing and avoid amputations. The primary objective was to find out the prevalence of DFUs getting infected with fungus and the secondary objective was to identify the appropriate methodology for the detection of the fungus in DFUs.

Materials and methods: This was a cross-sectional observational study carried out in a tertiary care hospital with a sample size of 60 DFUs. Microbiological analysis was done by swab culture and deep tissue culture. Observational data were collected and the significance level was statistically analyzed.

Results: In the present study, the prevalence of fungal infections in DFUs was 31.7%. Only fungal tissue was positive in 15%, the fungal swab was positive in 8.33%, and both tissue and swab were positive in 8.33%. All these patients were treated with antifungal treatment as per the culture report in addition to appropriate antimicrobial therapy.

Conclusion: A fungal culture should be done in all patients with non-healing DFUs. Both fungal swab and tissue culture testing should be advocated in patients with DFUs for better mycological evaluation. The addition of antifungal medications may provide better outcomes in selected cases.

## Introduction

Diabetes mellitus is the most common non-communicable disease in the world. Worldwide, over the last two decades, the prevalence of diabetes mellitus has increased from 30 million to 177 million. Based on current trends, it is estimated that more than 570 million will have diabetes by the year 2030 and >700 million by 2045 [1].

As per a recent study in India, 11.5% of adults more than 45 years have high blood sugar. Its prevalence is higher in elderly age groups, 60 years (26.1%) and above in urban and 9.3% in rural areas [2]. The prevalence was similar in both male (12%) and female (11.7%) populations [3]. Diabetic foot is defined as the presence of infection, ulceration, and/or destruction of deep tissues associated with neurological abnormalities and, various degrees of peripheral arterial disease (PAD) in the lower limb in patients with diabetes.

Diabetes patients have a 12%-25% risk of developing foot ulcers during their lifetime [4]. Diabetics who develop a foot infection have a 155-fold increased risk of amputation compared to those who do not have diabetes [5]. To date, the standard approach is to identify the bacterial infection and treat it with appropriate antibiotics ignoring the possibility of opportunistic fungal infections in diabetic foot ulcers (DFUs). However, candida species account for the most commonly isolated strains among the wide range of fungal strains in an infected DFU [6]. The ideal treatment of an infected DFU should encompass all the possible microbiological causes, to provide efficient and specific treatment to all such patients. In the present study, both swabs, as well as tissue culture, were taken for identification of fungal infection as swabs are often contaminated with normal skin flora or colonizers.

The primary aim of this study was to identify the prevalence of fungal infections in DFUs, secondarily the preferred method of fungal culture (tissue vs. swab), and to recommend appropriate treatment for better treatment outcomes.

## Materials and methods

A prospective study was conducted at the surgery department in collaboration with the microbiology department, Kalinga Institute of Medical Science, Bhubaneswar, a tertiary care hospital in Eastern India, from April 2018 to March 2020 after the approval of IRB and Institutional Ethical committee (KIMS/KIIT/IEC/126/2018).

A total of 60 patients admitted with DFUs from April 2018 to March 2020 were taken as the study population. Patients within the age group of 18-80 years were included in the study group; pregnant, nursing women and patients on chemotherapy/radiotherapy or prior antifungal therapy were excluded. In the evaluation of patients with DFU, the type and duration of diabetes, level of blood sugar, HBA1c, duration of ulcer, and history of prior antibiotic therapy were noted. Foot ulcers were classified according to Wagner’s grade of DFU. In all patients, fungal tissue and swab cultures with relevant patient specifics were noted in a master chart.

Sample collection/Culture and mounting

Before taking a culture from the depth of the ulcer the wound surrounding was cleaned thoroughly with 10% povidone-iodine followed by sterile normal saline. Culture swabs were taken from the depth of the ulcers by a cotton-tipped applicator measuring around 0.5 cm x 0.5 cm and kept in routinely used normal saliva-containing sterile containers. Punch biopsy forcep was used to take tissue from the depth of a particular ulcer under the aseptic technique. A tissue sample was collected, sealed, labeled, and sent to the microbiology lab within 1 h to detect the strains of fungal pathogens.

Laboratory procedures

The tissue sample was subjected to the following microscopic examination before the inoculation in culture media.

1) Microscopic examination of the KOH prepared tissue sample: tissue was kept on a slide with the addition of 10% KOH at 37°C for 2 h and then examined under the microscope.

2) Microscopic examination of Gram-stained tissue smear: a smear was obtained on a clean and dry slide from the crushed tissue sample and was dried, fixed by heating, and stained with Gram stain. After proper staining, it was examined under oil immersion for identification of Gram-positive budding cells and pseudohyphae, specific for Candida infection. 

Method of the culture of tissue sample

A culture media of Sabouraud’s dextrose agar (SDA) with chloramphenicol antibiotic was used for culturing the tissue sample. Four such culture tubes were prepared and tissue samples were inoculated. Cycloheximide (actidione) was added to two tubes out of four tubes. Culture tubes containing both chloramphenicol and cycloheximide were incubated at 25°C-30°C. The addition of chloramphenicol inhibits the growth of bacteria whereas cycloheximide inhibits the growth of contaminant saprophytic fungi. Cultures were examined twice a week for 4-6 weeks. 

Mounting of fungal growth

Once the growth was identified on the culture medium it was mounted using lactophenol cotton blue (LPCB) for staining filaments and spores of the fungus. Sometimes LPCB mounting disturbs fungal morphology during the process, so slide culture was added in most of the cases to protect the intact morphology of the isolated fungi. 

Statistical analysis

All the qualitative parameters were represented with frequency and percentages. To compare the differences between the two mean values, an unpaired t-test was applied and a p-value less than 0.05 was considered to be significant. The statistical software, SPSS 21.0 (IBM, Armonk, NY) was used for the analysis of the data. 

## Results

A total of 60 patients with diabetic foot were taken for the study. Anthropometric parameters such as age, gender, duration of diabetes and biochemical parameters such as fasting blood sugar (FBS), post prandial blood sugar (PPBS), glycated hemoglobin (HbA1c), and albumin were recorded. The correlation of incidence of fungal infection with different variables such as age, duration of diabetes, HbA1c, FBS, PPBS, and albumin was analyzed and represented in Table 1. A significant correlation was observed in the association of fungal infection with albumin (Table 1).

**Table 1:** Correlation of prevalence of fungal infection in diabetic foot ulcer with variables, i.e. age, duration of diabetes mellitus (in years), HbA1c, FBS, PPBS, and albumin. p-value < 0.05, considered as significant NS, not significant; HS, highly significant; HbA1c, glycated hemoglobin; FBS, fasting blood sugar; PPBS, postprandial blood sugar; DM, diabetes mellitus

Variables	Prevalence of fungi	Mean	p-value
Age (years)	Positive	57.63 ± 11.31	0.913
	Negative	57.95 ± 8.19	
Duration of DM (years)	Positive	7.89 ± 6.14	0.832
	Negative	7.51 ± 6.71	
HbA1c	Positive	10.56 ± 2.81	0.487
	Negative	10.03 ± 2.74	
FBS (mg/dL)	Positive	182.63 ± 100.53	0.499
	Negative	164.56 ± 82.08	
PPBS (mg/dL)	Positive	267.63 ± 154.76	0.479
	Negative	240.2 ± 88.93	
Albumin (g/dL)	Positive	3.05 ± 0.46	0.014
	Negative	2.72 ± 0.46	

**Figure 1:**
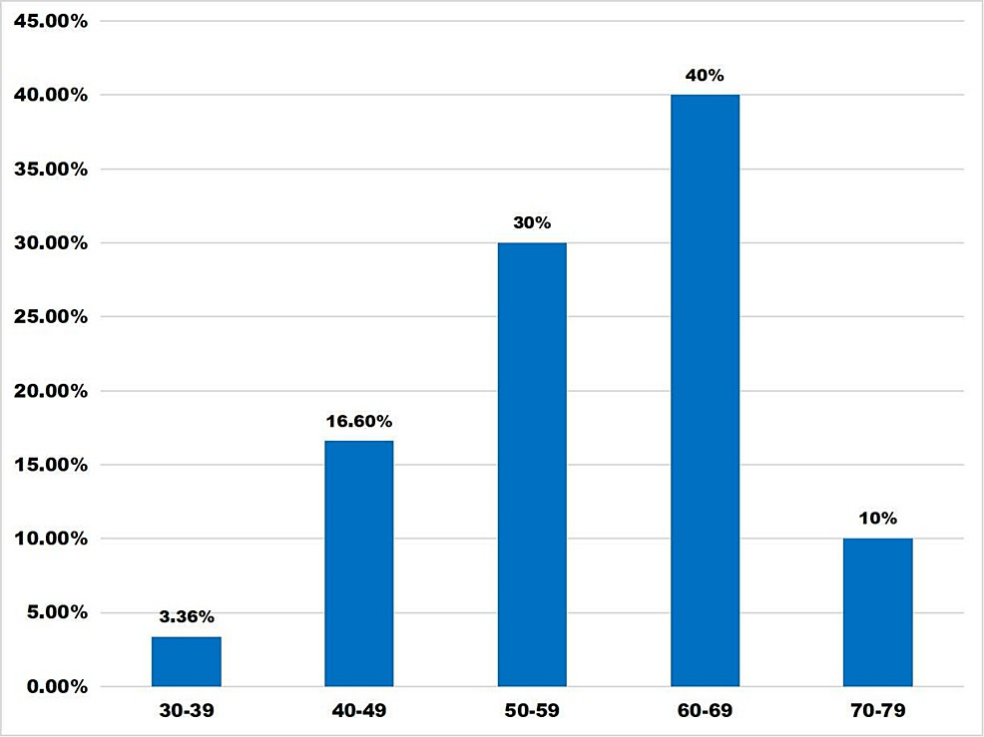
Age distribution (ranging from 30 to 80 years) in DFU patients. X axis: age ranging from 30 to 80 years Y axis: percentage of study population DFU, diabetic foot ulcer

In this study population, a maximum number of patients were males (50 males and 10 females) with a male and female ratio of 9.1. Most of the patients (40%) were observed in the age group of 60-69 years followed by 50-59 years. The average age was 57.85 years ± 9.1 with a minimum age of 35 years and maximum age of 70 years (Figure 1). 

**Figure 2:**
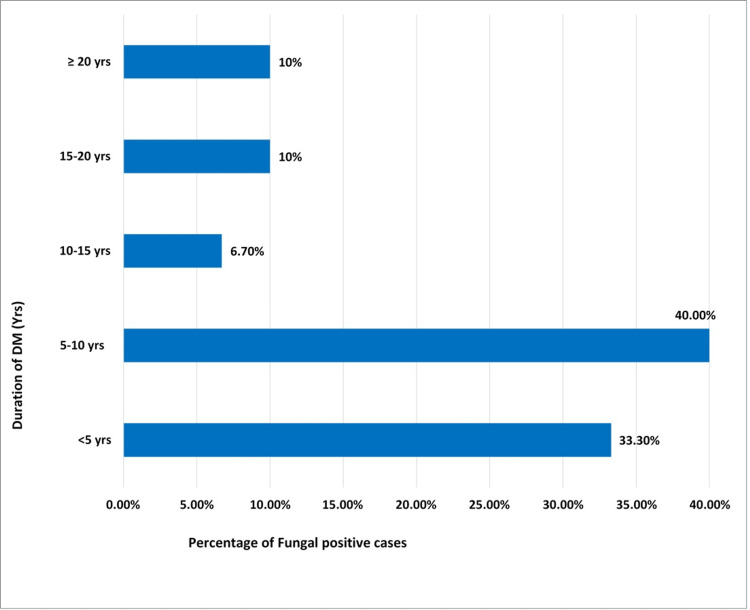
Representation of fungal cases with duration of diabetic in years.

**Figure 3:**
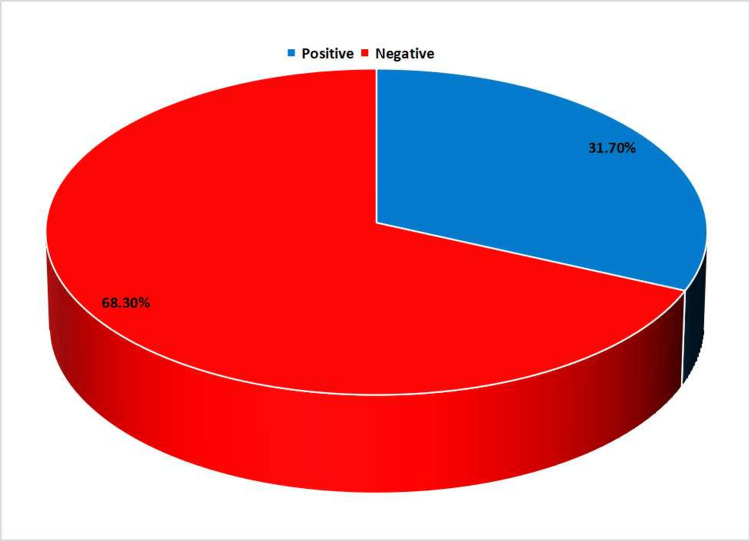
Prevalence of fungal infections in DFU, the prevalence of fungal infection was 31.7%. DFU, diabetic foot ulcer

The incidences of DFUs were more common in males in comparison to females 9:1. The duration of diabetes in maximum patients (40%) was 5-10 years (Figure 2). The mean duration of diabetes was 7.8 years ± 6.14 in the case of patients with positive fungal infection and 7.5 years ± 6.71 in the case of patients with no fungal infection. Prevalence of fungal infection was observed in 31.67% (19/60) cases of DFU (Figure 3).

**Figure 4:**
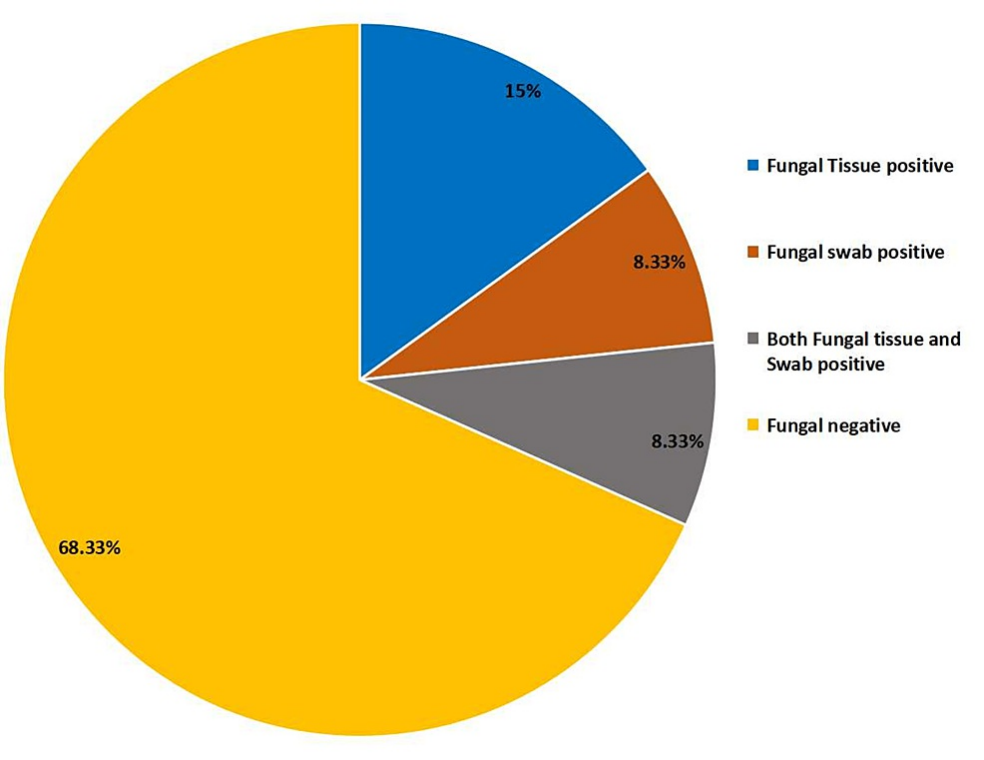
Prevalence of fungal infections in DFU on the basis of type culture. Maximum fungal positive cases were in the tissue culture. DFU, diabetic foot ulcer

**Figure 5:**
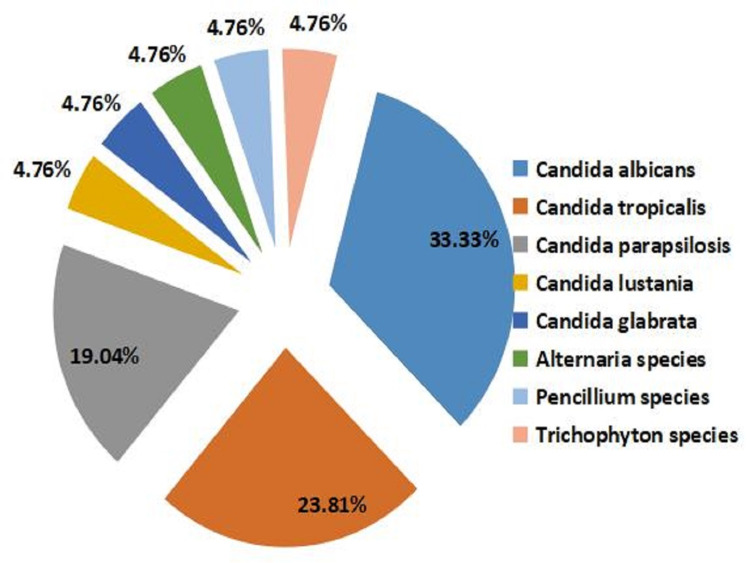
Different fungal isolates identified from study population.

Fungal tissue positive was observed in 15% (9/60) of patients; fungal swab positive was observed in 8.33% (5/60), and in 8.33% (5/60) both fungal tissue and swab were positive (Figure 4). Figure 5 shows the identification of fungus by KOH mounting and Gram staining. 

**Figure 6:**
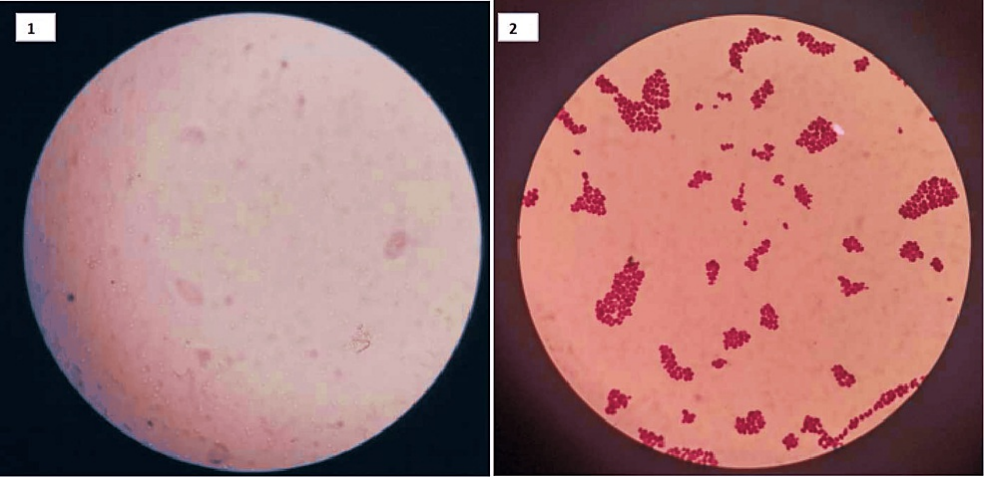
1. KOH mount for fungal identification. 2. Gram staining of fungal organism.

**Figure 7:**
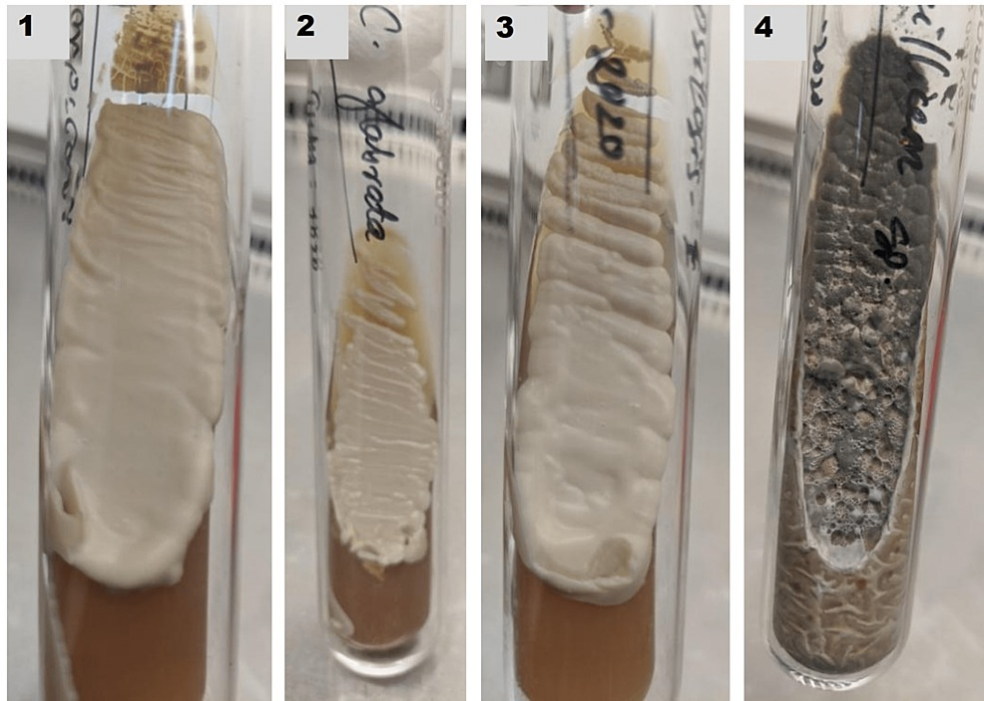
Candida and Penicillium species growth in SDA medium. 1 – *Candida tropicalis; *2 –* Candida glabrata; *3 – *Candida parapsilosis; *4 – *Penicillium species* SDA,Sabourauds dextrose agara

The most common fungal isolates were *Candida albicans* (33.33%) followed by *Candida tropicalis* (23.81%) and *Candida parapsilosis* (19.04%) (Figure 6). Figure 7 shows the Candida growth and Penicillium species in Sabouraud’s dextrose agara (SDA) medium. 

**Figure 8:**
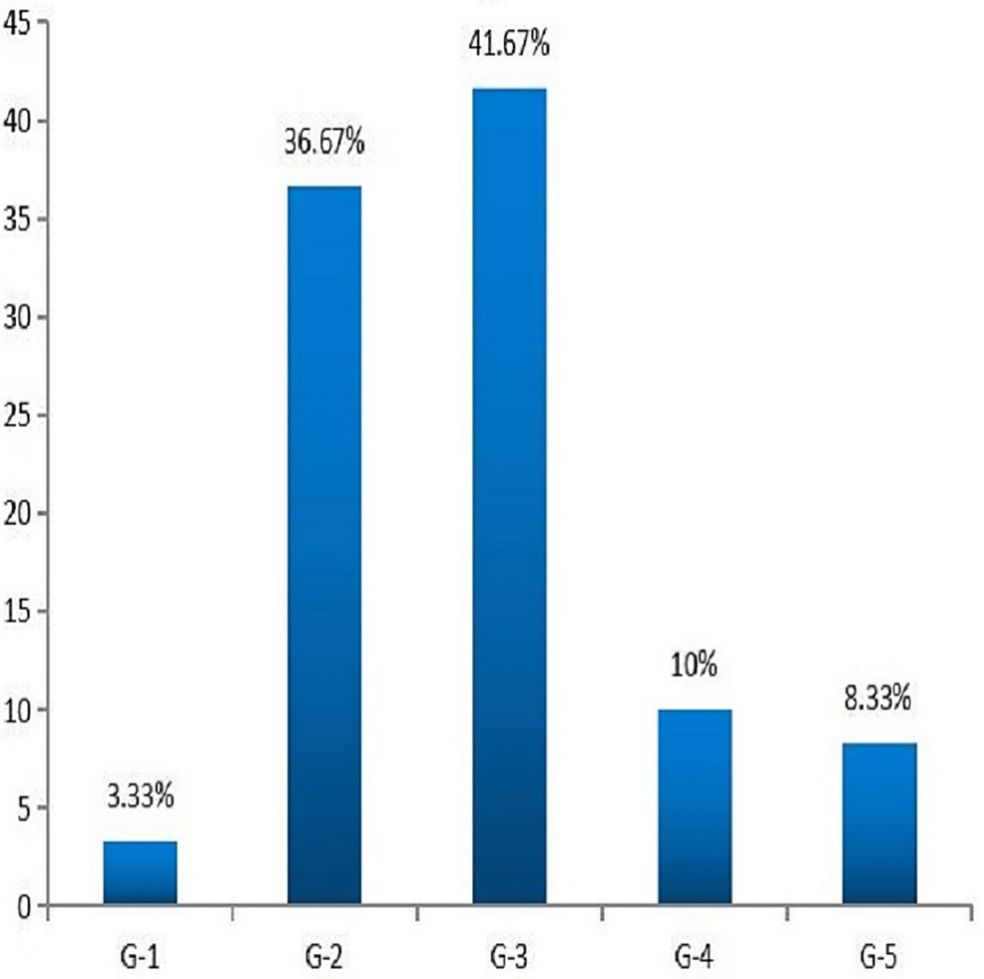
Classification of fungal infection based on Wagner's grades, most of DFU were observed in grades 2 and 3. DFU, diabetic foot ulcer

Most of the patients in the study were having Wagner’s grade 2 (36.67%) and grade 3 (41.67%) type of DFU and the maximum fungal isolates were identified in grade 2 (36.84%) and grade 3 (42.1%) (Figure 8). 

## Discussion

The patient care for diabetic foot ulceration is complex as the wound infections are polymicrobial leading to progression into deeper tissue ending with lower-limb amputations as a life-saving measure. Dermatophyte infections of toe web spaces provide an environment for subsequent colonization by bacteria. Sometimes dermatophyte infection can get exacerbated in an occlusive webspace environment leading to damage to the stratum corneum which promotes the overgrowth of commercial bacteria resulting in maceration and itching of the involved site. 

A total of 60 patients with diabetic foot were taken for observation, where maximum cases (40%) were seen in mostly 60-69 years of age. It is mostly seen in elderly patients due to long-standing diabetes with associated neuropathy and vasculopathy. Males are more affected because of greater outside activity and increased predisposition to trauma. There is a good correlation of DFUs with certain factors like gender, old age, poor glycemic control, prolonged disease duration with associated neuropathy, and vasculopathy [7]. 

Both swab and tissue biopsy are done routinely to assess the microbiological profile of infection, but tissue biopsy has the advantage of not getting prone to superficial contamination. International Working Group on Diabetic Foot (IWGDF) proposed to obtain culture for tissue rather than swab for better yield [8]. The results of the current study also reflected more towards tissue 15% for tissue positive, 8.33% for swab positive, and 8.33% for both fungal tissue and swab. The most widely used Wagner Classification System (sometimes referred to as Merritt-Wagner) was developed in the 1970s and comprises six ulcer grades, ranging from 0 to 5. This system assesses ulcer depth and the presence of osteomyelitis or gangrene [9]. The majority of our patients had grade 3 Wagner's ulcers (41.67%); the risk factors were mostly associated with lack of feeling in the foot, poor circulation, foot deformities, irritation (such as friction or pressure), and trauma, as well as the duration of diabetes. 

In the present study the incidence of *C. albicans* (33.33%), *C. tropicalis* (23.8%), and *C. parapsilosis* (19%) were maximum compared to other microbes. As reported by Missoni et al., tissue biopsy specimens were positive for fungus in 22 diabetic patients having clinical incidence of fungal infection. The fungal isolates were identified as *C. parapsilosis* (45.5%) followed by *C. tropicalis* (22.7%), *C. albicans* (9.1%), and *C. glabrata* (9.1%) [10-11].

Varsha et al. reported in their study that *C. albicans* was the most common fungal isolates (42.85%) in DFUs [11-12]. El-Nagar et al. in their study isolated *C. albicans* in 40.1% of 120 diabetic patients. In the study by Bansal et al., out of 103 patients with DFUs, superficial swab culture revealed fungal growth in 9% of cases [13]. Our study shows fungal isolation of 31.7% with isolation of fungi from swabs as 16.7% and from tissue as 23.3%. The discrepancy between the two studies may be due to better yield in tissue culture and also because of a limited sample size.

Fungal infections in the foot can be superficial infections or more complicated deep tissue infections. It is commonly affected by Candida species and *Fusarium solani*. The incidence of fungal infection was directly related to the duration of diabetes mellitus. Patients with a mean duration of diabetes of 7.7 years were having fungal infections [14]. In this study group, the mean duration of diabetes in the fungal positive patients was 7.89 years ± 6.14. There is a close association of diabetes foot ulcer with the duration of diabetes as reported by Al-Rubeaan et al. [15], whereas our study did not show any significant correlation between the fungal infection and duration of diabetes. 

Poor glycemic control was another common feature in diabetic patients with fungal culture positivity, who had an average fasting glucose (mg/dL) level of 182.63 mg/dL and a postprandial glucose level of 164.56 mg/dL. In the present study the mean HbA1c level of the patients with positive fungal culture was 10.56 ± 2.81 and that of fungal positive swab culture and fungal positive tissue culture were 9.62 and 11.31, respectively. Elevated glucose level (glycosylated hemoglobin HbA1C >9%) as one of the risk factors for ulceration has been reported by other authors [16]. Both duration and poor gylcemic control contribute to diabetic foot complications. Results revealed that fungal infections were more common in DFUs of longer duration.

The present study shows the mean albumin in all the fungal positive patients was 3.05 g/dL ± 0.46, fungal swab culture 3.09 g/dL, and fungal positive tissue culture 3.04 g/dL. A significant correlation was observed between fungal infection and albumin level. Many studies have not observed the association between albumin and fungal infections in DFU. So, more studies are required for the analysis of this association.

Large-scale studies are required for the evaluation of the role of fungal culture and its treatment in the healing of DFUs in order to avoid complications and amputation.

## Conclusions

In conclusion, infection of DFU is polymicrobial, mixed infection of both bacterial and fungal. Fungal infection is often ignored but potentially fatal and may lead to foot amputation if left untreated. Fungal culture should be added to the treatment protocol in all long-standing DFUs and it does not heal in spite of adequate antimicrobial and antibacterial therapy and appropriate care by concerned speciality experts. Both fungal swab and tissue culture testing should be advocated in patients with DFUs for better mycological diagnosis.

Furthermore, the present study includes only a small group of patients. So, further studies are required for a better analysis of the need for routine employment of fungal culture in patients with DFU to identify fungal infections and the addition of antifungal medication to the existing management protocol.

## References

[1] Powers AC, Niswender KD, Carmella EM (2018). Diabetes mellitus: diagnosis, classification, and pathophysiology. Harrison's Principles of Internal Medicine, 20e.

[2] Mishra NL, Gaikwad R, Bapat SV (2021). Diabetes among senior citizens more prevalent in urban India: LASI report. DownToEarth.

[3] Ramachandran A, Snehalatha C (2009). Current scenario of diabetes in India. J Diabetes.

[4] Andersen CA, Roukis TS (2007). The diabetic foot. Surg Clin North Am.

[5] Jia L, Parker CN, Parker TJ (2017). Incidence and risk factors for developing infection in patients presenting with uninfected diabetic foot ulcers. PLOS One.

[6] Subha KS, Shivaprasad H, Lakshmidevi N. (2013). Isolation and identification of pathogens from diabetic foot infections from K.R hospital, Mysore. Int J Dev Res.

[7] Kaabi J M Al, Maskari F Al, Zoubeidi T (2014). Prevalence and determinants of peripheral neuropathy in patients with type
2 diabetes attending a tertiary care center in the United Arab Emirates. J Diabetes Metab.

[8] Lipsky BA, Aragón-Sánchez J, Diggle M (2016). IWGDF guidance on the diagnosis and management of foot infections in persons with diabetes. Diabetes Metab Res Rev.

[9] Swezey L (2019). Swezey L. Diabetic foot ulcer classification systems. WoundEducators.com. https://woundeducators.com/diabetic-foot-ulcer/.

[10] Belicza M, Missoni E (2005). Candida infections in diabetic foot ulcers. Diabetol Croatica.

[11] Varsha T. Kalshetti, Rahul W, Bothikar ST (2017). Study of fungal infections in diabetic foot ulcer. Indian J Microbiol Res.

[12] EL-Nagar EL-Nagar (2018). Fungal diabetic foot infections. Egypt J Med Microbiol.

[13] Bansal E, Garg A, Bhatia S (2008). Spectrum of microbial flora in diabetic foot ulcers. Indian J Pathol Microbiol.

[14] Raymundo M, Mendoza MT (2002). The microbiologic features and clinical outcome of diabetic foot infections among patients admitted at UP-PGH. Phil J Microbiol Infect Dis.

[15] Al-Rubeaan K, Al Derwish M, Ouizi S (2015). Diabetic foot complications and their risk factors from a large retrospective cohort study. PLOS One.

[16] Lavery Lawrence A, David G, Armstrong SA (1998). Practical criteria for screening patients at high risk for diabetic foot ulceration. Arch Intern Med.

